# Intramuscular stimulation of tibialis anterior in human subjects: the effects of discharge variability on force production and fatigue

**DOI:** 10.14814/phy2.13326

**Published:** 2017-08-07

**Authors:** Michael Leitch, Rachael Brown, Vaughan G. Macefield

**Affiliations:** ^1^ School of Medicine Western Sydney University Sydney New South Wales Australia

**Keywords:** Discharge variability, FES, intramuscular stimulation, motoneurones

## Abstract

Continuous intramuscular stimulation of tibialis anterior (TA) was used to test the hypothesis that irregular trains of stimuli can increase force production and offset the magnitude of fatigue when compared with a continuous train of regular stimuli at an identical mean frequency (19 or 24 Hz). To achieve this, tungsten microelectrodes were inserted into the muscle belly into the motor point of the tibialis anterior muscle of able‐bodied individuals (aged 19–50) and stimulated at current intensities ranging from 5 to 7 mA. The motor point was stimulated with a continuous train of regular stimulation at either 19 or 24 Hz (*n* = 11) or until the force declined below 25% of the peak force at the onset of stimulation. For the first seven subjects, no fatigue was exhibited, and thus, we simply compared the forces generated by the regular and irregular segments of the continuous train (120 sec for each segment). For four additional subjects, we delivered a higher frequency train (24 Hz) that elicited some fatigue. Once the force had declined below 25% of the initial peak force (which took between 140 and 210 sec), the continuous irregular train was integrated. Interestingly, for those subjects who exhibited muscular fatigue, force always began to rise again once the irregularity was incorporated into the continuous regular train of stimulation at the identical mean frequency (24 Hz). We conclude that incorporating irregularity into continuous trains of stimuli offers a significant advantage to the human neuromuscular system during both fatigued and nonfatigued states and could offer benefits to therapies such as functional electrical stimulation (FES).

## Introduction

Damage to the central nervous system from stroke or spinal cord injury can result in a loss of motor function and/or somatic sensation. There are a range of therapeutic techniques that may be implemented in order to promote faster recovery. One commonly used therapy is functional electrical stimulation (FES) or functional neuromuscular stimulation, in which electrical stimuli are delivered to paralyzed muscles via surface electrodes. This therapy has shown to beneficial in increasing contractile strength (Carnstam et al. 1977; Stein et al. 2002), improving muscle tone (Gerrits et al. 2002) and improving energy efficiency during walking (Taylor et al. 1999) when volitional control has been lost due to injury. One major limitation of FES therapy is the rapid fatigue of muscle fibers (Nathan 1984; Shields 1995). The frequencies delivered during FES therapy are often very high, exceeding stimulation frequencies at which the force–frequency relationship has been previously established for human motor units (Westling et al. 1990; Thomas et al. 1991a; Macefield et al. 1996; McNulty et al. 2000). Indeed, it has been shown that microstimulation of single motor axons supplying the long finger flexor muscles at frequencies above 30 Hz does not increase force production (Fugelvand et al. 1999), Yet FES frequently stimulates at frequencies above 30 Hz. Finally, it is well documented that the human nervous system discharges with a significant variability ~25% (Macefield et al. 2000; Vaillancourt et al. 2002, 2003), yet current FES trains utilize regular (constant‐frequency) trains of stimulation that exhibit *zero* discharge irregularity.

Conventionally, there are two main approaches during FES therapy, in an attempt to maintain force when it declines due to fatigue – increasing the intensity of the current or increasing the frequency of the pulses. However, fatigue is a multifactorial process and cannot always be prevented by these two methods (Binder‐Macleod and Barker 1991). A previous study conducted by our laboratory emphasized the advantages of incorporating physiological variability (irregularity) into trains of stimuli. This was achieved by microstimulating single human motor axons with both short (10 pulses, 8–24 Hz) and long‐lasting (850 pulses, 18 Hz) trains of irregular stimuli that emulated the firing of volitionally activated motor units. It was shown that discharge variability augments contractile responses and reduces the magnitude of fatigue for the toe extensors (Leitch and Macefield 2014, 2017). The purpose of this study was to test the hypothesis that incorporating irregularity into a *continuous* frequency train of stimuli delivered to multiple motor units of the tibialis anterior muscle would enhance force production when compared with a train of identical mean frequency with *zero* variability. A secondary objective was to observe whether discharge variability has any effect on those muscles that have exhibited minor fatigue. As aforementioned, we have shown these to be effective in single motor units and we wanted to test whether the same increases were seen when activating multiple motor units at the motor end point.

## Methods

### Generating the irregular trains

By recording the firing patterns of the tibialis anterior muscle, we were able to generate irregular trains of stimuli, which incorporate both short (high‐frequency) and long (low‐frequency) interspike intervals. An intramuscular tungsten microelectrode was inserted into the muscle belly of tibialis anterior in a single subject performing isometric ankle dorsiflexions. The subject was asked to sustain ~30% maximal voluntary contraction for 70 sec. The mean frequency for this train was 19.3 ± 8.7 Hz, with a discharge variability of 32.3% (S.D/mean × 100). Instantaneous frequencies ranged from 5.7 to 151.5 Hz. The inter‐spike intervals exhibited by this train were used to create an irregular train that emulated the firing of volitionally activated motoneurones.

Successful experiments were conducted on seven male and three female subjects (19–51 years) under the approval of the Human Research Ethics committee of the University of Western Sydney. Subjects provided informed written consent. The participants reclined in a chair, with the knee flexed to approximately 120°. The foot was fixed onto a rigid footplate with the ankle at 120° and a Velcro strap was attached over the dorsum of the foot, proximal to the metatarsophalangeal joints, so as not to interfere with dorsiflexion of the digits. The leg was supported by a vacuum cast to prevent any movement during the procedure. The anterior compartment of the leg was palpated to locate tibialis anterior (TA), and a 2‐mm diameter surface probe was used to electrically stimulate the muscle belly to identify a motor point, defined as the site requiring the least current to evoke a twitch. An insulated tungsten microelectrode (FHC, Maine, USA) was then inserted directly into this site and manually adjusted while delivering constant current cathodal pulses (0.02–1.0 mA, 0.2 msec, 1 Hz) to elicit a twitch. EMG was measured via surface Ag/AgCl electrodes placed over TA (BioAmplifier, ADInstruments, Sydney) and filtered (10 Hz–1 kHz) and recorded (2 kHz sampling) on a computer‐based data acquisition and analysis system (PowerLab 16 SP hardware and LabChart Pro 7 software, ADInstruments, Sydney). Force (DC‐100 Hz; 200 Hz sampling) was measured using a highly sensitive force transducer (Model 1030; UFI, Morro Bay, USA) located over the medial aspect of the foot at the first metatarsal; the transducer was oriented so as to record the optimal dorsiflexion/intorsion force vector. An appropriate intramuscular site was established when the EMG and force responses were clear and well defined. The muscle was first stimulated with a set of eight pulses at the threshold current (5–7 *μ*A, 0.2 msec, 1 Hz). This was done purely to ensure a clear response was seen from the TA. Force responses and instantaneous frequency were measured using the Peak Parameters feature of LabChart (ADInstruments, Sydney).

After the twitch parameters had been measured, a regular train of stimuli was delivered for 120 sec, followed by an irregular train of identical mean frequency for a further 120 sec (continuously). This allowed us to compare the peak forces generated by the irregular and regular segment of the continuous train. The peak forces were compared in 5‐sec intervals to determine any changes in force responses between the two stimuli. For seven subjects, the mean frequency was 19 Hz, while for four, we used a higher frequency (24 Hz) in attempt to promote fatigue. For these subjects, stimulation at 24 Hz was continued until force declined by 25% of the force at the onset of stimulation, which took between 140 and 210 sec. At this point, the irregular train was given for 120 sec to measure the effects of the irregular train on force production in a fatigued state. During delivery of all trains, there was *no relaxation time* between the regular and irregular trains of stimuli – i.e. the stimulation was continuous.

Statistical analysis was performed using Prism 6 software (GraphPad Software, San Diego, USA). To test our hypothesis that the irregular trains produced greater forces than the regular trains over the same mean frequencies, paired one‐tailed t‐tests were performed. We compared the total peak forces produced by the irregular and regular stimuli at 5‐sec intervals throughout the train of stimulation. This allowed a direct comparison of the forces generated by the two types of trains.

## Results

**Figure 1:**
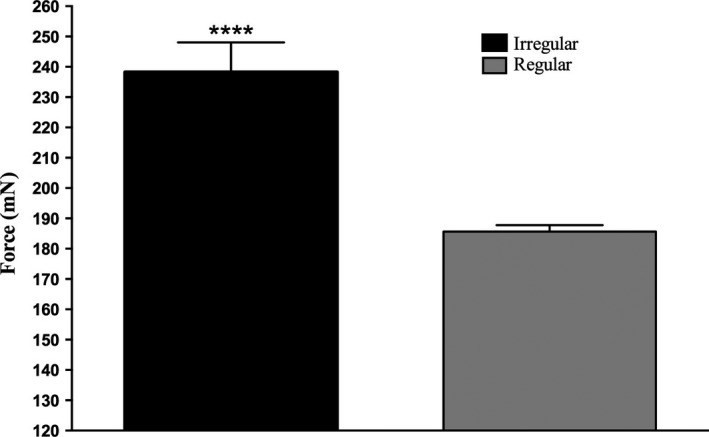
Peak forces generated during intramuscular stimulation of tibialis anterior (*n* = 10). Irregular trains generated higher contractile forces than regular trains over the same mean frequencies; *P* < 0.0001.

This study utilized intramuscular stimulation to activate motor units in the tibialis anterior muscle of human subjects (*n* = 11). For all stimulation sites, the peak forces (± SEM) for the regular trains were 188.4 ± 63.3 mN. This was significantly lower than the peak forces produced by the irregular trains (238.3 ± 81.3 mN; *P* < 0.0001; paired t‐test), as seen in Figure 1. By incorporating discharge variability, the contractile responses were either increased back to initial peak responses when the muscle exhibited a decline in force, or the contractile responses increased from a plateau when the muscle displayed no decline in the force.

**Figure 2:**
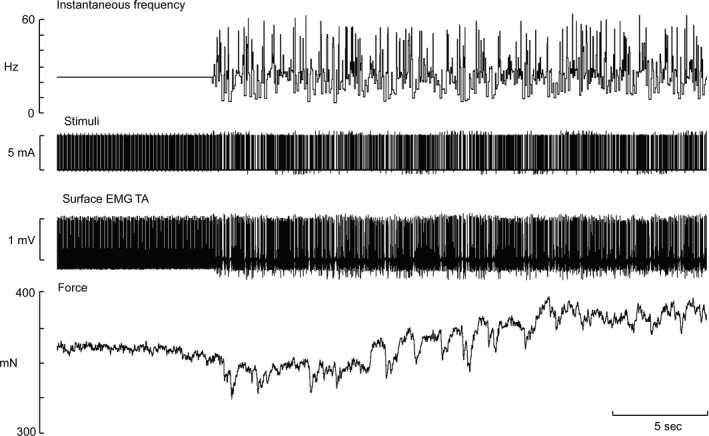
Raw data from a single subject during intramuscular stimulation of tibialis anterior at 5 mA. This subject was given the higher frequency train (24 Hz) to promote fatigue. Instantaneous frequency is shown in the top trace. The bottom trace shows the decline in force and the rapid recovery once the irregularity is integrated into the train.

Experimental records from one subject are shown in Figure 2. The irregularity can be seen in the instantaneous frequency channel (top) and in the EMG channel (middle). It can be seen that when the peak force declined by ~25%, then the irregular stimulation pattern commenced. This resulted in an augmented force production over the last 85 sec of stimulation. Peak force was 462 mN at the onset of the regular stimulation train, falling to 364 mN at 205 sec, before reaching a peak force of 472 mN at the conclusion of the irregular stimuli.

**Figure 3:**
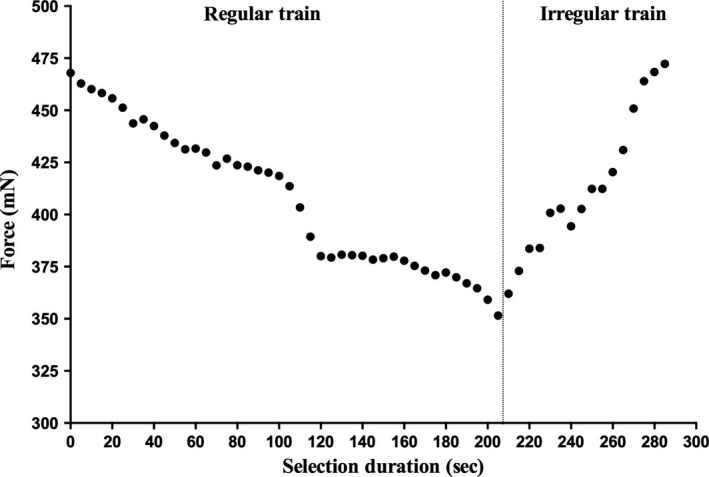
Data from a single subject during intramuscular stimulation of tibialis anterior. A continuous train of regular stimuli was delivered at 24 Hz for 205 sec, at which force had fallen by ~25%. At the vertical line, irregularity (variability) was incorporated at the same mean frequency. Force rose to a peak of 472 mN at the conclusion of stimulation.

Data from another subject are shown in Figure 3. The peak values shown here have been averaged to obtain the peak forces over 5‐sec intervals. The initial peak force (±S.D) after the first 5 sec was 462.9 ± 4.6 mN, which declined by ~25% to 364.7 ± 1.5 mN after 205 sec of continuous stimulation. Once the irregular stimulation paradigm commenced (at 210 sec), the peak force increased from 362 ± 7.9 mN to a peak of 472.3 ± 4.9 at the conclusion of irregular stimulation. There was a statistically significant difference between irregular and regular stimuli (*P* = 0.001; paired t‐test).

**Figure 4:**
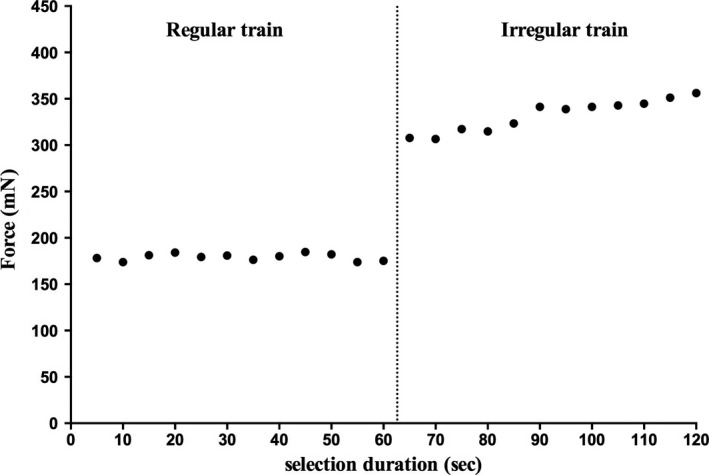
Data from a single subject during intramuscular stimulation of tibialis anterior. A continuous train of stimulation was given at 19 Hz for 120 sec; 60 sec are shown on either side of the transition between regular and irregular stimulation (vertical line). Peak force was 175 mN at the conclusion of the regular train and 365 mN at the conclusion of the irregular train of stimulation.

If there was no decrease in the force after 120 sec of continuous regular stimulation, then it was assumed that we were activating a group of fatigue‐resistant units. Figure 4 shows the peak forces, sampled over 5 sec, for a different subject during stimulation with both the regular and then irregular train. We compared 120 sec of force generation either side of the transition between the two types of trains. It can be seen that force plateaued for this subject and did not show any signs of fatigue. Peak force was 178.3 mN after 5 sec of stimulation and 175.2 mN at 120 sec. After the variability was incorporated peak force increased significantly to 307.9 mN at 65 sec to a maximum of 356.1 mN at the conclusion of stimulation (120 sec).

## Discussion

This study has shown that continuous trains of stimuli that incorporate irregularity produce greater forces during intramuscular stimulation of motor units at the motor end plate of tibialis anterior when compared with regular trains over the same mean frequencies, even when fatigue was present. When force began to decline, irregular trains were able to increase the force toward the initial level of force that was generated at the onset of the regular train and sustained this force until the conclusion of stimulation. Moreover, for those motor units that did not fatigue, irregular trains increased forces above those produced for the regular trains. This emphasizes the importance of (1) the effects of irregular stimulation patterns on force production and (2) on reducing the magnitude of fatigue for long continuous trains of stimulation.

### Mechanisms for augmentation of force

Initial “doublets” – two pulses with an interval of 10 msec – preceding a train of stimuli result in higher forces, both in whole muscle and during activation of single motor units (Binder‐Macleod and Barker 1991; Macefield et al. 1996). It has been suggested that the initial augmentation in force is due to extra release of calcium ions and not prolonged release of calcium or slower reuptake (Duchateau and Hainaut 1986). It has also been shown that during the fatigued state, the available myoplasmic calcium is reduced and thus the muscle undergoes a decline in force (Westerblad et al. 1993). Therefore, a viable explanation for this phenomenon is that when calcium availability is depleted, i.e. in a fatigued state, incorporating a few short interspike intervals (irregularity) could promote the release of more calcium, therefore resulting in augmentation of force.

Another possible explanation may be due to muscle stiffness, required to build tension during contractions; it has been shown that in the fatigued state muscle stiffness decreases (Wilson et al. 1994). During isotonic movements, in which the muscle is constantly shortening, there is a decrease in muscle stiffness (Curtin and Edman 1994). However, when a short doublet is given in isometric conditions, where no shortening is required, the doublet is said to rapidly take up tension and force is able to build up more effectively (Binder‐Macleod and Lee 1996). In this study, measurements were also taken during isometric conditions. Therefore, we suggest that incorporating short and long interspike intervals – as seen with the irregular stimulation patterns used here – increases muscle stiffness, which contributes to the buildup of tension.

### Limitations of FES therapy

Irrespective of the benefits, one major limitation of FES therapy is rapid fatigue of muscle fibers when using external stimulation (Nathan 1984; Shields 1995; Jones 1996; Peckham and Knutson 2005). There are a number of reasons as to why paralyzed muscles fatigue so rapidly. First, because patients with chronic SCI have muscles predominately composed of fast‐fatigable (type II) fibers that are have poor fatigue resistance (Burnham et al. 1997; Klein et al. 2006), this is certainly not something that we can resolve in this study. Unlike voluntary contractions, electrically stimulated contractions are thought to recruit larger more fatigable motor rather than smaller and more fatigue‐resistant ones. When stimulating whole muscle via surface electrodes, larger motor units are preferentially activated before smaller motor units (Sinacore et al., 1990; Llewellyn et al. 2010). Larger motor units have greater axon diameters and supply more muscle fibers than smaller motor units (Henneman et al. 1965), thereby generating greater forces. However, this becomes problematic as they predominately supply fast‐fatigable muscle fibers (Henneman 1957; Burke et al. 1973) thus rapid fatigue occurs due to a reversal of recruitment order (Trimble and Enoka 1991; Heyters et al. 1994; Binder‐Macleod et al. 1995; Feiereisen et al. 1997). While this issue is not addressed in this study, the use of irregularity has still been shown to reduce the effects of fatigue and increase contractile responses when activating a group of motor units within TA. This irregularity takes advantage of the “catch‐like property” which is known as the force augmentation produced by the inclusion of a short, high‐frequency doublet, at the onset of a subtetanic train of stimuli (Binder‐Macleod and Barker 1991; Bevan et al. 1992; Callister et al. 2003). Until now, studies that have examined the effects of the “catch‐like property” in whole muscle have only examined its effects at the onset of a train of stimuli (Binder‐Macleod and Lee 1996; Binder‐Macleod et al. 1997). Studies have examined variable trains of the thenar muscles with variable trains of stimuli using supramaximal stimulation (Indurthy and Griffin, 2007), but no study has incorporated irregularity throughout trains of stimuli while stimulating the motor end point of human leg muscles.

### Advantages of incorporating variability into trains of stimuli

Most advances in FES have involved improvements in technology, programming, and implementation (Lee et al. 1999). The long‐term goal, however, is to help restore motor function for those who have been injured, and the biggest problem at present is rapid fatigue. Integrating for uses in FES therapy may be beneficial if there is less fatigue exhibited by the active muscles. The order in which the motor units are recruited is not something that is resolved in this study, and not something that can be easily resolved in the future. However, we have previously shown that incorporating physiological variability into brief trains of stimuli (10 pulses) is effective in augmenting force and reducing fatigue (Leitch and Macefield 2014). This study shows that this also holds true for long‐lasting trains of stimuli to multiple motor units.

## Conclusions

Irregular trains of stimuli offer an advantage to the neuromuscular system by augmenting force production and reversing the effects of fatigue during a 120‐sec train of stimulation. Current FES therapies utilize regular (constant‐frequency) trains of stimuli that incorporate zero variability, usually at frequencies that are higher than those at which motor units discharge during voluntary contractions. Both of these factors promote muscle fatigue, which is a major limitation of FES therapies. We propose that incorporating discharge irregularity may improve the current stimulation patterns utilized during FES, thereby improving the short‐term therapeutic effects.

## Conflict of Interest

None declared.
